# *In vivo* evidence of the prevents DSS-induced colitis of *Lactiplantibacillus plantarum* L15

**DOI:** 10.3389/fmicb.2022.1028919

**Published:** 2022-10-06

**Authors:** Zengbo Wang, Liu Yang, Hongwei Tang, Kangyong Zhang, Qingxue Chen, Caihua Liu, Yanan Guo, Minghao Li, Zengwang Guo, Bailiang Li

**Affiliations:** ^1^Food College, Northeast Agricultural University, Harbin, China; ^2^Key Laboratory of Dairy Science, Ministry of Education, College of Food Science, Northeast Agricultural University, Harbin, China

**Keywords:** *Lactiplantibacillus plantarum*, inflammatory response, intestinal integrity, gut microbiota composition, SCFAs, NF-κB signaling pathway

## Abstract

Ulcerative colitis (UC) is challenging to treat and severely impacts patients and families. A previous study reported immunomodulatory and reduction of pro-inflammatory properties for the *Lactiplantibacillus plantarum* L15. This study aimed to analyze the preventive properties and mechanistic actions in an *in vivo* colitis model. The histopathological alteration, inflammation cytokines, and intestinal barrier function were analyzed. Subsequently, the cecal gut microbiota contents and products from different groups were detected. Finally, gene expressions related to the NF-κB signaling process were evaluated. *L. plantarum* L15 significantly decreased disease activity index (DAI), myeloperoxidase activity (MPO), pro-inflammatory cytokine (TNF-α, IL-1β, and IL-6) level, and increased weight change, colon length, and production of inflammation-suppressing cytokines. Furthermore, this strain supplementation substantially increased ZO-1, Occludin, and Claudin-1, and MUC2 mRNA expression levels with a corresponding decrease in serum lipopolysaccharide and D-lactic acid contents. In addition, *L. plantarum* L15 improved gut microbiota composition and increased short-chain fatty acid (SCFAs) in the colon content, which significantly reduced the transfer of NF-κB p65 to the nucleus. Our findings provide a theoretical basis for *L. plantarum* L15 as a preventive candidate for UC.

## Introduction

Ulcerative colitis (UC) is a swelling condition affecting the colorectal region, with clinical manifestations like ulcer and abdominal pain (Colombel and Mahadevan, 2017). Increased UC incidences in developing countries have been reported recently, making it a subject of research interest (Mak et al., 2020). Although the exact causes of UC are still unclear, recent research findings indicate that imbalances in immune responses, mucosal barrier damage in the intestinal region, environmental and genetic indices are possible drivers (Xavier and Podolsky, 2007). DSS is a water-soluble sulfated polysaccharide that is toxic to gut epithelial cells and disrupts the integrity of the mucosal barrier, causing an acute colitis characterized by rectal bleeding, diarrhea, ulcerations, and granulocyte infiltrations (Okayasu et al., 1990), resembling clinical and histological features of human UC. Thus, they are widely used to assess the pathogenesis of UC.

Bioinformatics insights have become increasingly relevant in evaluating gut microbiota components and harnessing them in disease therapy. Gut microbiota disruptions could cause UC onset, a process that can ultimately destroy the human mucosal barrier, an integral pathogen-fighting hub (Manichanh et al., 2012). The intestinal mucosal barrier comprises four parts: mechanical, chemical, biological, and immune (Ying et al., 2020). The intestinal epithelial barrier is constructed by a single layer of columnar intestinal epithelial cells connected through tight junctions to form a complete intestinal epithelial barrier (Anderson and Itallie, 2009). There is a connection between gut microbiota and the UC with phenomenal advances in sequencing techniques. Several studies have supported the hypothesis that the gut ecosystem is crucial in UC onset and development (Castro-Dopico and Clatworthy, 2019; Zeng et al., 2021; Ahmed et al., 2022).

Although conventional UC medications (aminosali-cylates, steroids, immunomodulators, and biological drugs) have proven effective in some respect, they are also accompanied by deleterious side effects such as infection, fever, allergy, and hepatorenal syndrome (Guidi et al., 2011; Bernstein et al., 2016; Feuerstein et al., 2016; Celiberto et al., 2018). Thus, the need to explore other effective and less harmful alternatives has been the focus of many research works. *Lactiplantibacillus plantarum* strains are one of the most valuable probiotics, and their beneficial effect on colitis has been proved in numerous animal experiments and clinical trials (Kan and Nanno, 2008; Zhai et al., 2019). Currently, many reports have illustrated that *L. plantarum* strains significantly affect the risk of IBD (Choi et al., 2019; Liu et al., 2020a). For example, *L. plantarum* −12 reversed gut dysbiosis in Balb/c mice by improving intestinal inflammation (Sun et al., 2020). *L. plantarum* AR326 could ameliorate induced colitis by enhancing tight junction protein expression levels, suppressing pro-inflammatory cytokines (Wang et al., 2019a). *L. plantarum* with high conjugated linoleic acids-synthesized ability could alleviate UC by blocking the NF-κB signaling process (Liu et al., 2020a). *L. plantarum* KLDS 1.0386 could improve DSS-induced UC by up-regulating mRNA expression levels of aryl hydrocarbon receptor (AHR) *via* the IL-22/STAT3 signaling pathway (Shi et al., 2020). In addition, *L. plantarum* NCU116 with high EPS production could regulate the structural modification of the gut microbiota and enhance epithelial barrier functions in some *in vivo* inflammation studies (Zhou et al., 2019, 2021). These studies focused on the ameliorative effect of *L. plantarum* strains on DSS-induced murine colitis. Nevertheless, there is insufficient data on the study of *L. plantarum* strains preventing UC development.

In a previous study, *L. plantarum* L15 has been reported to have immunomodul-atory and inflammation-suppressive characteristics (Yu et al., 2020). This study analyzes the preventive role and mechanistic actions of *L. plantarum* L15 intervention in a colitic model *in vivo* by evaluating its effects on DSS-induced colitis, histopathological alteration, inflammation cytokines, and intestinal barrier function. Subsequently, the cecal gut microbiota contents and their products from different groups were detected. Finally, the expression of genes related to the NF-κB signaling pathway was evaluated.

## Materials and methods

### Study strain

*Lactiplantibacillus plantarum* L15 strain was isolated from Yak yoghurt, which was obtained from Gansu Province, China, identified, and cultured following a recent study (Yu et al., 2020). The strain was grown in MRS broth at 37°C for 18 h, harvested (6,000 g, 10 min, 4°C), and washed twice with phosphate-buffered saline (PBS, pH 7.4). Bacterial cells were re-suspended at 1 × 10^10^ CFU/ml in PBS.

### Animals and experimental design

**Figure 1:**
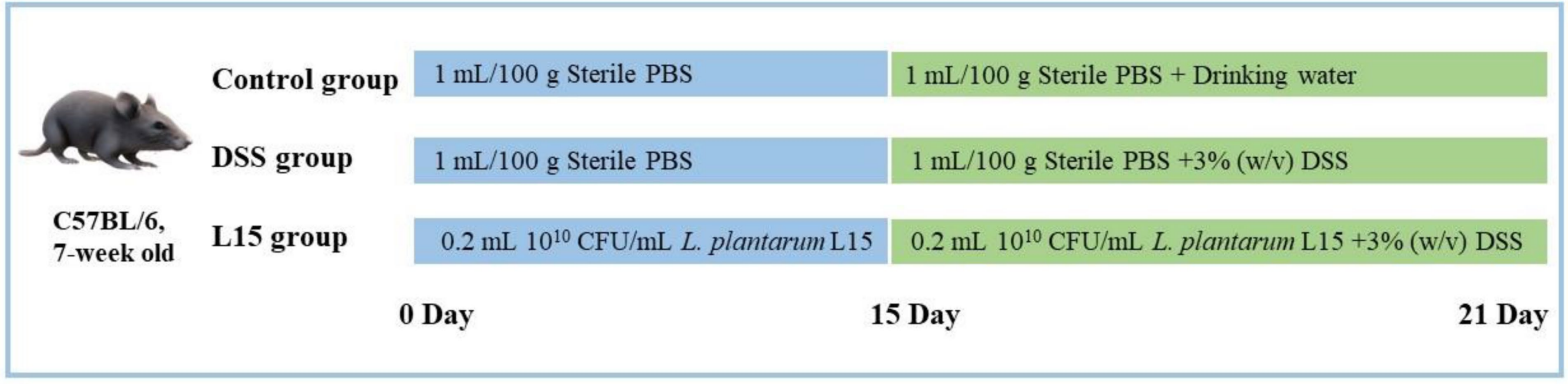
Animal model experimental design.

7-week old C57BL/6 mice (*n* = 36) were supplied by the 2nd Affiliated Hospital of the Harbin Medical University (Harbin, China). They were acclimatized as recently described by Shi et al. (2021). The Northeast Agricultural University Animal Care approved all animal experiments and protocols (Authorization No: NEAUEC2001121). Mice were randomly and equally divided into three groups (*n* = 12) after 1 week acclimatization period. From days 1 to 21, the control group (NC) and the DSS intervention group (DSS) were fed with sterile PBS (1 ml/100 g body weight/day). In addition, the *L. plantarum* L15 group (L15) was given 0.2 ml of *L. plantarum* L15 suspension (1 × 10^10^ CFU/ml). On days 15–21, drinking water was supplied to the DSS and L15 groups with 3% (w/v) DSS, while the NC group was provided only with drinking water. The experimental arrangement is depicted in Figure 1.

### Colitis evaluation

The body weight, stool consistency and blood were measured daily throughout the experiment following a previously described procedure (Cooper, 1993). Disease activity index (DAI) was scored based on an average score of body weight change, stool consistency and hemoccult bleeding from a previous scoring system (Murthy et al., 1993; Camuesco et al., 2004). The mice are executed at the end of the experiment, the colon tissue is removed and the length of the colon is measured. The colon and intestinal contents are stored at −80°C. Blood is obtained from the hearts of the mice. After a period of resting, the serum is obtained by centrifugation (3,000 *g*, 20 min) and stored at −80°C.Test kits for myeloperoxidase analysis obtained from the Nanjing Bioengineering Co. Ltd. were used to obtain sample MPO levels.

### Histological analysis

Refer to previous research (Wang et al., 2019a). Briefly, A 4% paraformaldehyde solution was used to preserve the distal colon samples for 24 h. Afterward, they were placed in paraffin, and thin-layered sections (5 μm) were marked with hematoxylin and eosin (HE) solution before analysis. The samples were observed at ×40 and ×200 magnification and the severity of colonic histological injury was scored using a scoring system taking into account the severity of inflammation (from 0 to 3 as the maximum score), crypt damage (from 0 to 5 as the maximum score) and ulcerations (from 0 to 3 as the maximum score) as previously described (Laroui et al., 2012). All data were examined in six replications.

### Colon tissues cytokines

These were determined following the procedure of Shi et al. (2021). Briefly, the supernatant samples to be analyzed were obtained from homogenized and centrifuged colon tissues. ELISA test kits purchased from the Nanjing Bioengineering Institute were used to determine TNF-α, IL-1β, IL-6, and IL-10 levels.

### Serum D-lactic acid and lipopolysaccharide levels

Serum D-lactic acid and lipopolysaccharide (LPS) contents were analyzed using the ELISA kits purchased from the Bioengineering Institute in Nanjing, China based on the manufacturer’s instruction.

### Quantitative real-time PCR

The Total RNA Kit (Vazyme, Nanjing, China) was used to obtain the total RNA of the colon tissue of each mouse, and cDNA was obtained by reverse transcription using the GoScriptTM Reverse Transcription Mix kit (Promega, Madison, United States). The mRNA gene expression was carried out with a GoTaq R SYBR-Green qPCR Master Mix (Promega, Madison, United States). The Claudin1, Occludin, ZO-1, β-actin, and MUC2 primers synthesized by Sangon Biotech (Shanghai) Co., Ltd. are shown in Table 1. For normalization, β-actin was used as the housekeeping gene, and the 2^−ΔΔCt^ method was used for data analysis.

**Table 1:** Primer sequences for qPT- PCR.

Genes	Forward(5′-3′)	Reverse(5′-3′)
Claudin1	GCTGGGTTTCATCCTGGCTTCTC	CCTGAGCGGTCACGATGTTGTC
Occludin	TTGGCTACGGAGGTGGCTATGG	TTACTAAGGAAGCGATGAAGCAGAAGG
ZO-1	CATAAGGAGGTAGAACGAGGCATCATC	CGATCACCACCCGCTGTCTTTG
β-actin	GGTTGTCTCCTGCGACTTCA	TGGTCCAGGGTTTCTTACTCC

### Gut microbiota analysis

Following the manufacturer’s instructions, bacterial DNA in the cecal contents from the three groups was obtained using a QIAamp DNA stool mini kit (Qiagen, Dusseldorf, Germany). The V3-V4 region of the 16S rDNA was selected for generating PCR amplicons with the following primer order: forward primers: 5′-ACTCCTACGGGAGGCAGCAG-3′; reverse primers: 5′-GACTACHVGGGTWTCTAA-T-3′, and the Illumina Miseq platform (Illumina, Santiago, USA) was used for pyrosequencing. The analysis pipeline followed the procedure of Li et al. (2018). The PICRUSt software (model, country) was used to predict the functional potential of gut microbiota. Relative predicted abundance of MetaCyc pathways was calculated by dividing each pathway’s abundance by the sum of all pathway abundances per sample (Beaumont et al., 2020).

### Short-chain fatty acids analysis

This step was performed as described in the previous research (Cooper, 1993). First, 80 mg of cecal contents were treated using a HALO-F100 fecal analyzer, and 500 μl was mixed with crotonate monophosphate solution. After filtration, the supernatant was placed in a meteorological bottle. Gas chromatography (GC) analysis was then carried out as previously described.

### Western blotting

An extraction kit (Beyotime Institute of Biotechnology, Shanghai, China) was used to prepare the protein samples. Sealed SDS-PAGE protein isolates were transferred to the membrane following the procedure. Finally, the membrane was treated with an ECL kit to display antibody-specific proteins, and protein levels appearing as luminescent bands were quantified using the Gel-Pro Analyzer.

### Statistical analysis

In this study, the one-way ANOVA and GraphPad Prism 8.0.2 statistical software were used to analyze the obtained data, expressed as mean ± standard deviation (SD). In addition, *P* confidence levels at 5 and 1% levels were considered statistically significant.

## Results

### *Plantarum* L15 and DSS-induced colitis symptoms

**Figure 2:**
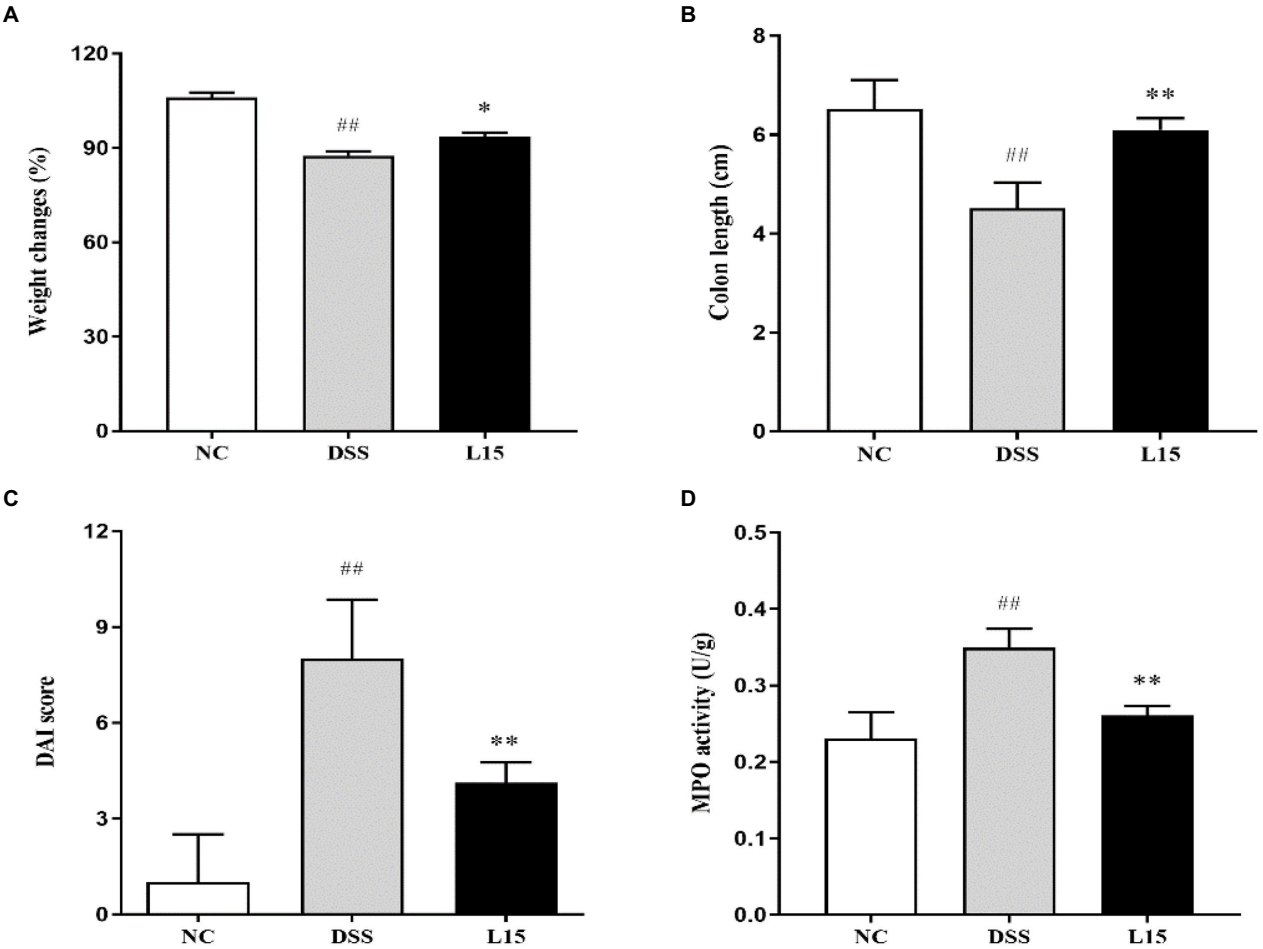
Results of *Lactiplantibacillus plantarum* L15 supplementation on colitis features triggered by DSS **(A)** Changes in body weight (%), **(B)** Colon length (CM), **(C)** DAI score, and **(D)** MPO activity (U/g). NC, normal control group; DSS, dextran sulfate sodium-induced colitis group; L15, Supplement *L. plantarum* L15 group. Data are presented as mean ± SD (*n* = 3). ^##^*p* < 0.01 and ^#^*p* < 0.05 vs. the NC group. **p < 0.01 and *p < 0.05 vs. the DSS group.

Our results show that mice weight changes and colon length in the DSS group were significantly (*p* < 0.05 and *p* < 0.01) lower than that of the NC group (Figure 2); however, the DAI and MPO of the mice in the DSS group were significantly higher (*p* < 0.01 and *p* < 0.01) than that of the control group (NC). Interestingly, these four indicators were substantially ameliorated (*p* < 0.05, *p* < 0.01, *p* < 0.01, and *p* < 0.01) following supplementation with *L. plantarum* L15, indicating that its inclusion could prevent UC conditions stimulated by DSS.

### *Lactiplantibacillus plantarum* L15 supplementation effect on colon histopathological alterations

**Figure 3:**
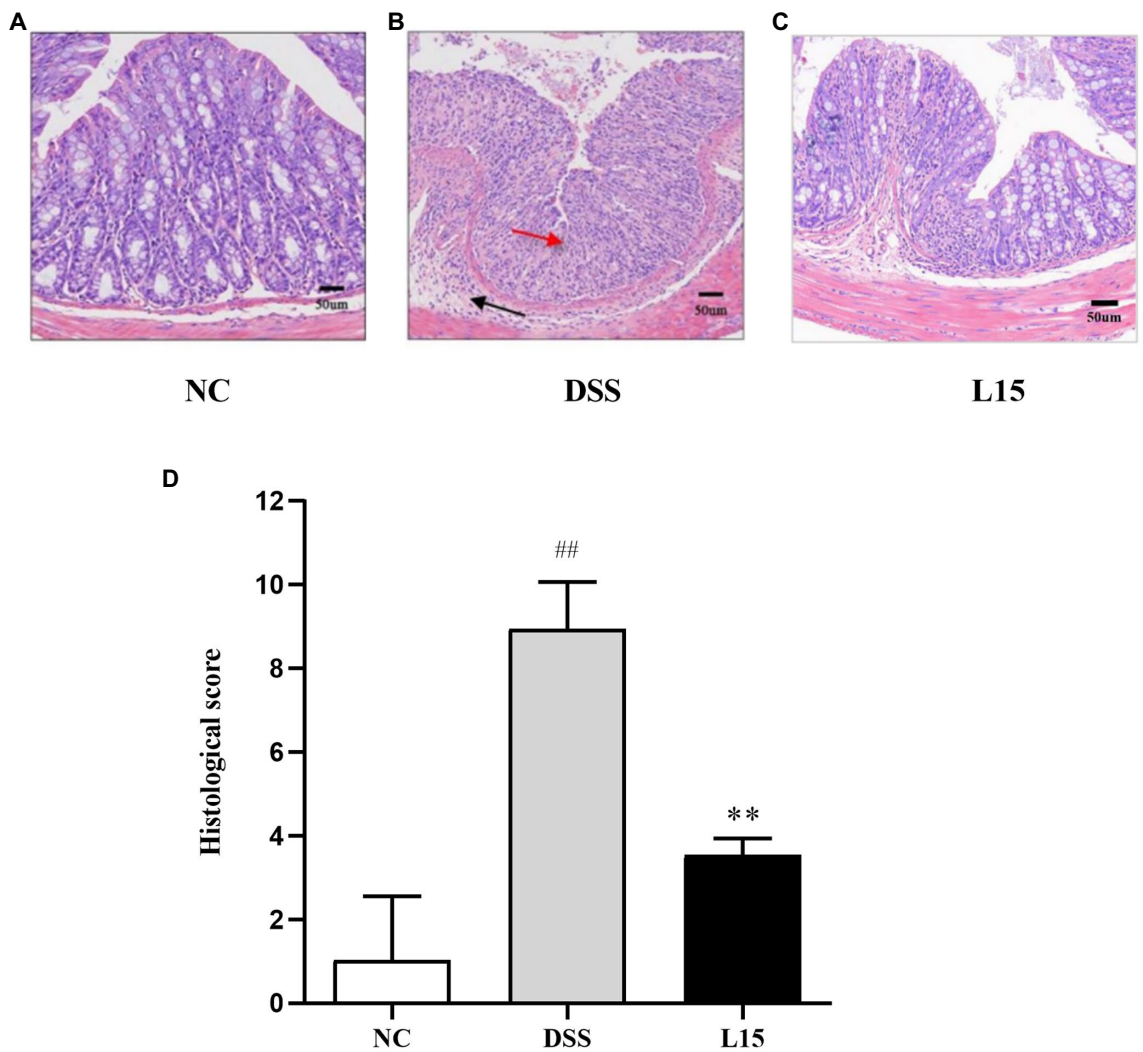
Effect of *L. plantarum* L15 supplementation on the mouse colon histological changes (H&E). **(A)** NC group (×40), **(B)** DSS group (×200), **(C)** L15 group (×200), **(D)** Associated histological scores. NC, normal control group; DSS, dextran sulfate sodium-induced colitis group; L15, Supplement *L. plantarum* L15 group. Data are presented as mean ± SD. ##p < 0.01 and #p < 0.05 vs. the NC group. **p < 0.01 and *p < 0.05 vs. the DSS group.

Histological analyses results indicate that NC group colon samples had a regular morphological layout with unruptured mucous membranes (Figure 3). As expected, samples from DSS-treated mice were characterized with tall columnar colonocytes on the surface and damaged long crypts. Moreover, weak goblet cell ruptures caused by inflammation and edematic submucosa were noticed. These characteristics were substantially reduced in the L15 group.

### *Lactiplantibacillus plantarum* L15 administration and colon tissue cytokines production

**Figure 4:**
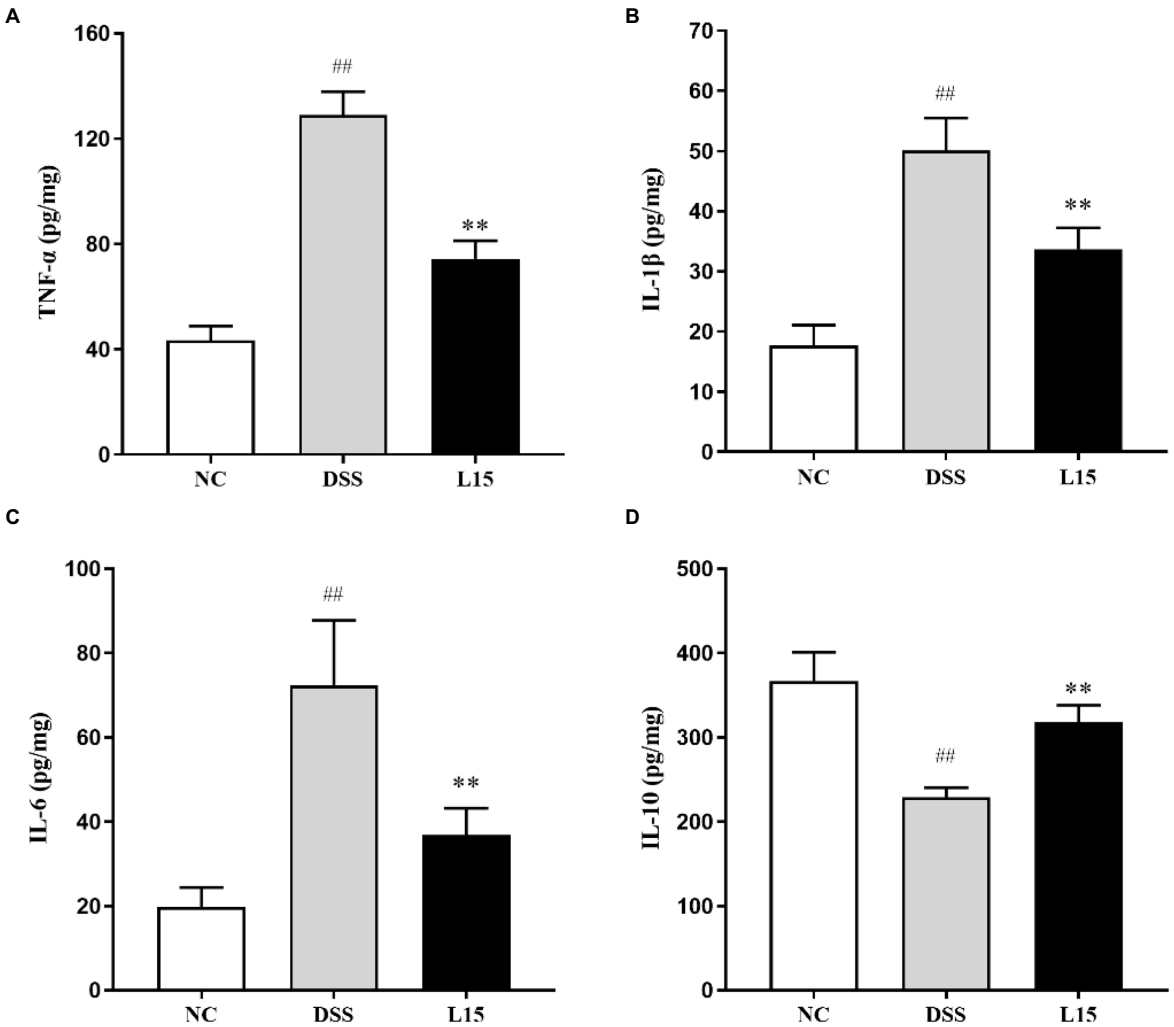
Effects of *L. plantarum* L15 supplementation on cytokine concentrations. **(A)** TNF-α, **(B)** IL-1β, **(C)** IL-6, and **(D)** IL-10. NC, normal control group; DSS, dextran sulfate sodium-induced colitis group; L15, Supplement *L. plantarum* L15 group. Data are presented as mean ± SD (*n* = 3). ^##^*p* < 0.01 and ^#^*p* < 0.05 vs. the NC group. **p < 0.01 and *p < 0.05 vs. the DSS group.

As shown in Figures 4A–C, a significant increase (*p* < 0.01) in TNF-α, IL-1β, and IL-6 levels in the DSS group compared with the control was found. However, there was a significant reduction (*p* < 0.05) in the TNF-α, IL-1β, and IL-6 levels of mice in the L15 group compared with the DSS group. Moreover, the level of IL-10 of the mice in the DSS group was significantly lower than that of the L15 group. The level of IL-10 was significantly increased (*p* < 0.01) in the L15 group as compared to the DSS group (Figure 4D).

### *Lactiplantibacillus plantarum* L15 inclusion and intestinal barrier function

**Figure 5:**
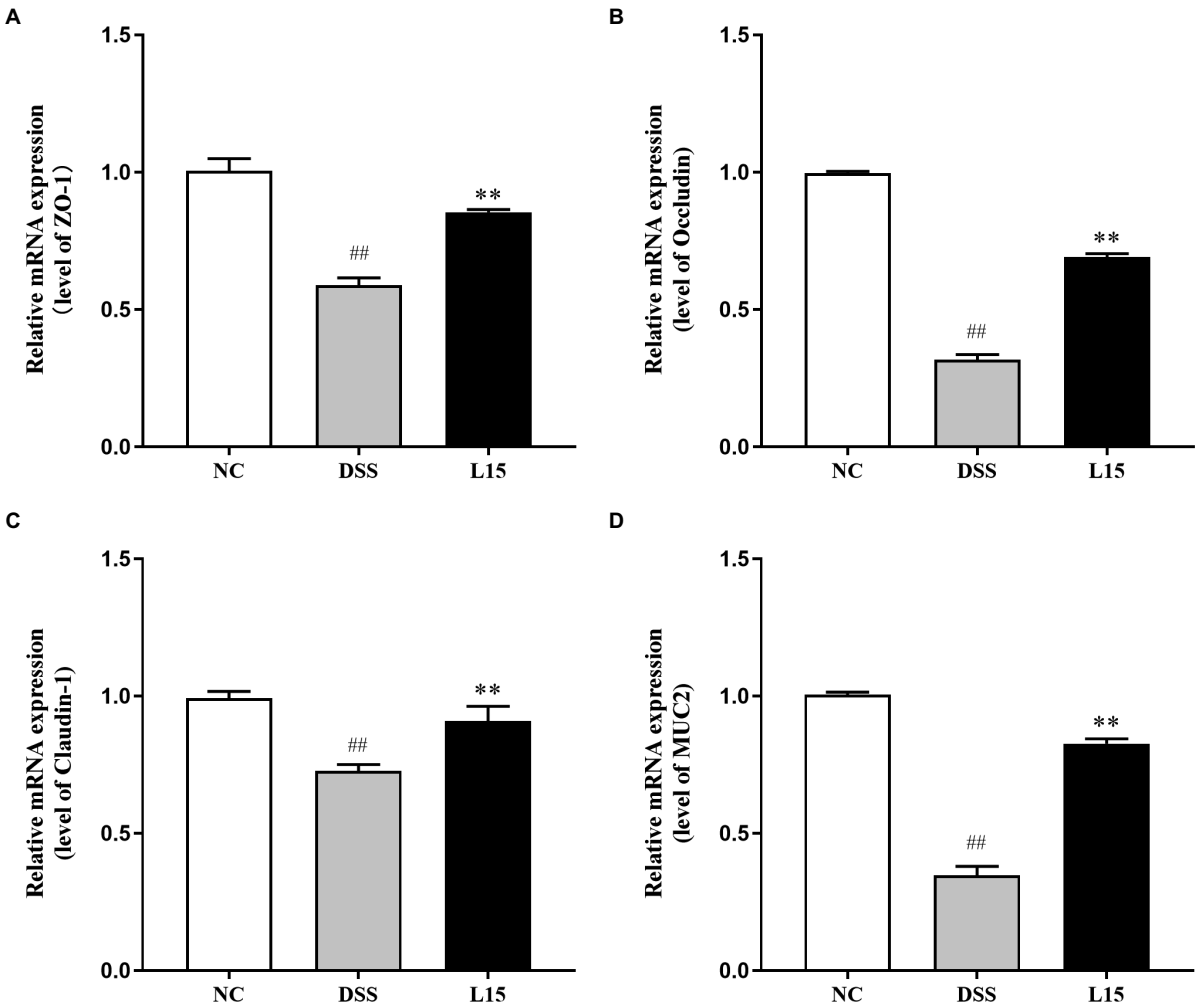
Effect of *L. plantarum* L15 supplementation on intestinal barrier related gene expression of colonic tissues. **(A)** ZO-1, **(B)** Occludin, **(C)** Claudin-1, and **(D)** MUC2. NC, normal control group; DSS, dextran sulfate sodium-induced colitis group; L15, Supplement *L. plantarum* L15 group. Data are presented as mean ± SD (*n* = 3). ^##^*p* < 0.01 and ^#^*p* < 0.05 vs. the NC group. **p < 0.01 and *p < 0.05 vs. the DSS group.

The mRNA expression levels of tight junction proteins and mucin in colonic tissues are reported in this study (Figure 5). The ZO-1, Occludin, Claudin-1, and MUC2 mRNA expression levels were considerably lowered following DSS inducement (*p* < 0.01), indicating that the barrier function was suppressed. Interestingly, *L. plantarum* L15 administration substantially up-regulated these parameters (*p* < 0.05), indicating that *L. plantarum* L15 inclusion had protective effects on epithelial integrity.

### *Lactiplantibacillus plantarum* L15 supplementation effect on serum contents

**Figure 6:**
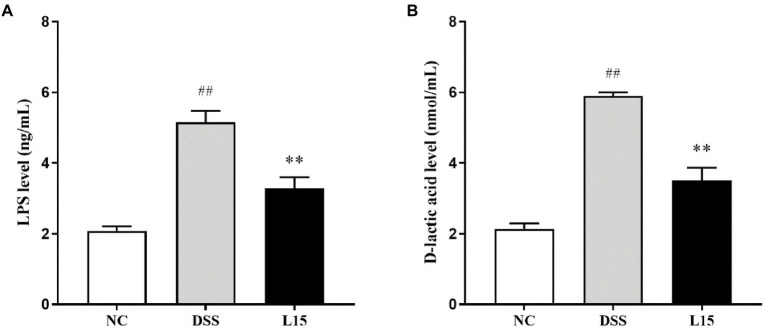
Effect of *L. plantarum* L15 supplementation on serum LPS **(A)** and D-lactic acid **(B)** levels. NC, normal control group; DSS, dextran sulfate sodiuminduced colitis group; L15, Supplement *L. plantarum* L15 group. Data are presented as mean ± SD (*n* = 3). ^##^*p* < 0.01 and ^#^*p* < 0.05 vs. the NC group. **p < 0.01 and *p < 0.0.05 vs. the DSS group.

In the present study, the serum contents investigated were the LPS and D-lactic acid concentrations. These parameters were elevated following DSS exposure (*p* < 0.01), while *L. plantarum* L15 inclusion substantially improved these two indicators (Figures 6A,B; *p* < 0.01).

### *Lactiplantibacillus plantarum* L15 and the gut microbiota composition

**Figure 7:**
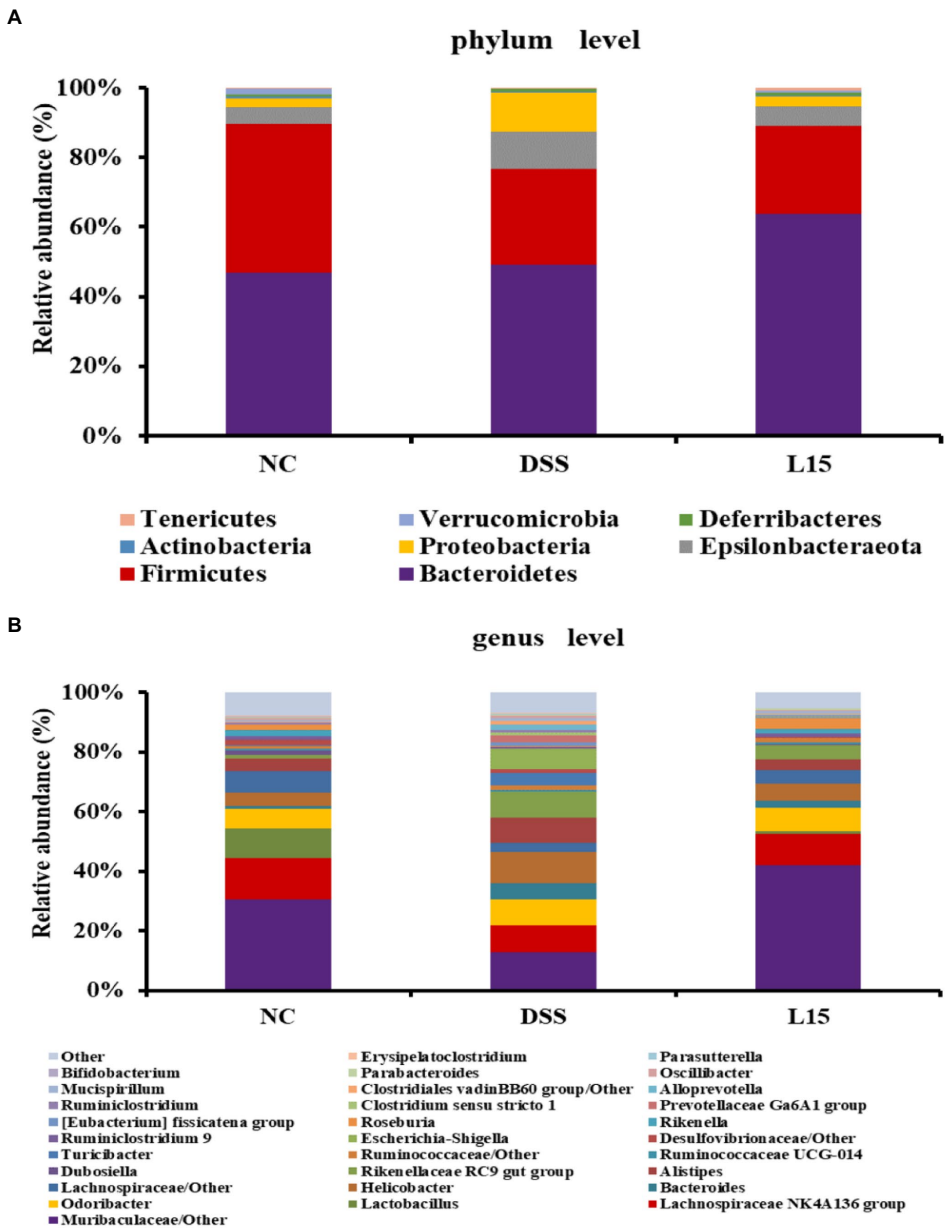
Gut microbial composition in analyzed mice fecal samples at the phylum **(A)**, and genus **(B)** levels. NC, normal control group; DSS, dextran sulfate sodium-induced colitis group; L15, Supplement *L. plantarum* L15 group.

The phyla and genera levels of microbial gut contents in the three study groups are reported (Figure 7). At the phylum level (Figure 7A), DSS induction raised Epsilonbacteraeota, Proteobacteria levels but diminished Firmicutes and Verrucomicrobia abundances than that in the NC group. However, *L. plantarum* L15 supplementation partially ameliorated these patterns. At the genus level (Figure 7B), *Muribaculaceae*, *Lachnospiraceae* NK4A136, *Lactobacillus*, *Rikenella*, and *Bifidobacterium* levels at the genus level were reduced in the DSS group when compared to that of the NC group. Interestingly, *L. plantarum* L15 supplementation normalized the disruptions in these genera. Furthermore, the pathogenic genera such as *Bacteroides*, *Helicobacter*, *Alistipes*, and *Escherichia-Shigella* increased in the DSS group, but these pathogenic genera were inhibited by *L. plantarum* L15. These observations suggest that *L. plantarum* L15 supplementation reshaped the gut microbiota structure to the pattern in the NC group.

### *Lactiplantibacillus plantarum* L15 and SCFAs production

**Figure 8:**
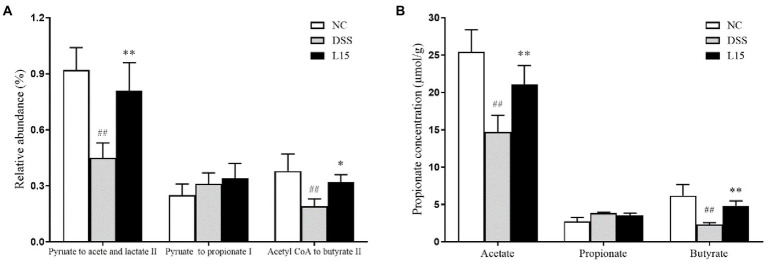
The relative abundance of pathways related to SCFAs production in the cecal contents was predicted using the PICRUSt **(A)**, and GC determined SCFAs production in the cecal contents **(B)**. NC, normal control group; DSS, dextran sulfate sodium-induced colitis group; L15, Supplement *L. plantarum* L15 group. Data are presented as mean ± SD (*n* = 3). ^##^*p* < 0.01 and ^#^*p* < 0.05 vs. the NC group. **p < 0.01 and *p < 0.05 vs. the DSS group.

Based on gut microbiota results, the PICRUSt software was used to further predict its potential to product SCFAs (Figure 8A). The DSS group showed lower acetic and butyric acid secretion levels than the control cohort (*p* < 0.05), but this improved after supplementation with *L. plantarum* L15. However, the potential propionate production did not show any significant difference across all groups. Thus, the GC method was employed to measure the SCFAs production in the colon to validate our results further. As shown in Figure 8B, the results of GC were in line with the predicted results. Thus, this strain could regulate SCFAs production *via* modulating gut microbiota.

### *Lactiplantibacillus plantarum* L15 treatment and the NF-κB pathway

**Figure 9:**
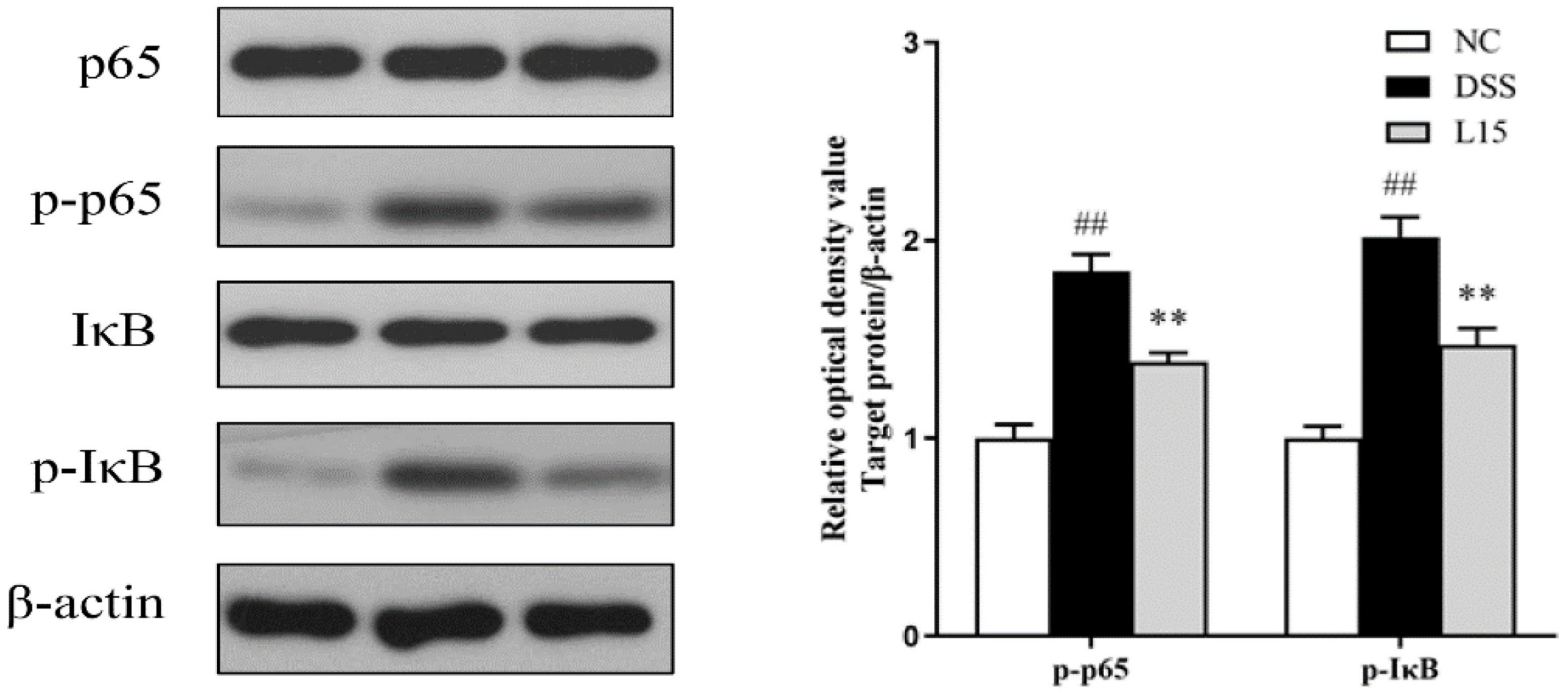
*L. plantarum* L15 treatment effect on the protein levels of NF-κB signaling pathway. NC, normal control group; DSS, dextran sulfate sodium-induced colitis group; L15, Supplement *L. plantarum* L15 group. Data are presented as mean ± SD (*n* = 3). ^##^*p* < 0.01 and ^#^*p* < 0.05 vs. the NC group. **p < 0.01 and *p < 0.05 vs. the DSS group.

Western blot analysis results indicate that the phosphorylation levels of NF-κB p65 and IκB increased markedly (*p* < 0.01) in the DSS group compared to the NC group (Figure 9). Interestingly, these levels were markedly suppressed by *L. plantarum* L15 supplementation (*p* < 0.01).

## Discussion

Ulcerative colitis is an acute inflammatory condition triggered by several factors, and its prevalence in younger people has increased in recent years. In addition, the WHO has noted that it has a high recurrence rate that presently has no effective therapy (Jb et al., 2013; Conrad et al., 2014; Parian et al., 2020). Interestingly, some trial sessions to assess the IBD-ameliorating potentials of *L. plantarum* have been suggested. Therefore, we examined the protective effect of *L. plantarum* L15 and the possible mechanisms involved in a colitic mice model.

The physiological and biochemical processes in UC have been investigated severally using the DSS-induced UC mouse model, as it gives a holistic picture of potential treatment options (Yhla et al., 2018). Changes in body weight and the presence of diarrhea and hematochezia symptoms have been used to measure DAI. Also, tissue damage and inflammatory responses have been measured by MPO levels (Sahu et al., 2016). Several studies have indicated that DSS-induced mice show reduced weight, have shortened colon regions, diarrhea, and increased DAI and MPO (Park et al., 2015; Shi et al., 2021). We observed that a 7-day DSS exposure had deleterious effects such as marked decrease in the weight changes and colon length and increased DAI and MPO. These trends were significantly reversed and colon damage repaired after *L. plantarum* L15 administration. These results were in line with the findings of Shi et al. (2021). Therefore, *L. plantarum* L15 supplementation showed good prevention on the symptom of UC induced by the DSS.

The increased secretion of pro-inflammatory cytokines under abnormal conditions climax in colon inflammation (Yuti et al., 2019). These include the TNF-αfactor implicated in intestinal mucosal damage, while other cytokine groups inhibited this process. A previous report indicated that cytokines luke Il-10 suppressed pro-inflammatory responses (Jesudas et al., 2019). After 7 days of DSS exposure, a considerable elevation and drop in pro-inflammatory and anti-inflammatory cytokines were observed in the present study. However, we found that *L. plantarum* L15 can effectively regulate the level of these cytokines, and this agrees with previous work by Sun et al. (2020). Therefore, *L. plantarum* L15 supplementation could prevent the UC induced by the DSS by regulating cytokines.

An integral part of the intestinal epithelial cell barrier is the tight junction., a complex matrix of transmembrane proteins, including peripheral membrane proteins (zonula occludens, Zo), transmembrane proteins (Occludin and Claudins), binding molecules (JAM), and intracellular regulatory molecules (kinases and actin; Stio et al., 2016). Tight junction damage can occur at the onset of colitis, making immune cells susceptible to inflammatory responses (Martens et al., 2018). The current study thus aimed to give insight into new ways of improving intestinal and immune homeostasis by preventing inflammatory reactions. In particular, the ZO-1 and occludin play complex roles in intestinal homeostasis by coordinating cell bypass barrier function and ensuring the optimal functioning of tight junction protein molecules (Buschmann et al., 2013). Claudins and MUC2 are other integral inflammation regulators whose activities could be useful in anti-inflammation therapy (Barmeyer et al., 2017). The raised mRNA expression of these proteins after treatment with L15 agrees with the observations of Liu et al. (2020b). Furthermore, the DSS group had higher serum D-lactic acid and LPS levels than the NC group. After *L. plantarum* L15 administration, these two indicators were restored to some extent. These levels were restored to normalcy following L15 supplementation, thus suggesting its potential role in improving intestinal integrity.

Findings from this study also indicated varying levels of specific phyla after treatment with DSS compared to the control (Figure 7A), an observation that agrees with earlier reports (Shi et al., 2020; Wang et al., 2020). Interestingly, *L. plantarum* L15 supplementation partially alleviated these imbalances. In the current study, the DSS group had lower relative abundance levels of beneficial microbes like *Lactobacillus* and *Bifidobacterium* at the genus level in the DSS treatment group, which were increased in the L15 group. For example, the *Muribaculaceae* has been reported to enable control of pro-inflammatory properties (Qi et al., 2017). It has been reported that the *Lachnospiraceae NK4A136* group and *Rikenella* are the SCFAs producer in gut microbiota with potential UC-ameliorating properties (Shi et al., 2017; Wang et al., 2019b). *Lactobacillus* and *Bifidobacterium* are known as probiotics, which can ameliorate UC (Liu et al., 2020a; Zhao et al., 2021). Furthermore, they can produce acetate and lactate, which can promote butyrate production (Chatelier et al., 2013). Furthermore, the DSS group had high levels of pathogenic bacteria groups like *Escherichia-Shigella*, *Helicobacter*, and *Alistipes* were higher in the DSS group compared with the control, while those were decreased in the L15-administered group. These gram-negative bacteria are involved in immuno-compromising processes that trigger the onset of UC (Mukhopadhya et al., 2012). In addition, the *Bacteroides* and *Alistipes* genera have been linked with LPS secretion, known to increase abdominal pain in IBS sufferers (Saulnier et al., 2011; Lorenzo et al., 2019).

Previous investigation has reported possible IBD treatment with the colonic content gut microbiota by SCFA production (Venegas et al., 2019). Based on gut microbiota results, the predicted relative abundances of acetate and butyrate production pathway analyzed by PICRUSt increased in the L15 group, supported by elevated acetate and butyrate levels determined by GC. In addition to protecting the intestinal mucosa and tight junction, SCFAs have been implicated in intestinal and immune homeostasis (Zhang et al., 2020). Furthermore, SCFAs have alleviated UC conditions by suppressing the NF-κB signaling pathway, closely linked with inflammatory cytokines and chemokines (Lührs et al., 2002; Venkatraman et al., 2003). The phosphorylation of IκBα and release of p65 into the nucleus are known pro-inflammatory triggers (Zhao et al., 2017a,b). In this study, we determined the genes expression levels associated with the NF-κB process by Western-blot. *L. plantarum* L15 supplementation could down-regulate NF-κB p65 and IκB phosphorylation levels, which could support the decreases of pro-inflammation levels and increase of anti-inflammatory concentration as aforementioned. Liu et al. (2020a) found that *L. plantarum* with high conjugated linoleic acids-synthesized ability could alleviate DSS-induced colitis by inhibiting the NF-κB signaling pathway. The results indicated that *L. plantarum* L15 prevented the DSS-induced colitis by modulating gut microbiota structure to increase SCFAs levels, which could suppress inflammation and improve the intestinal barrier *via* the NF-κB signaling pathway.

## Conclusion

The need to explore new UC therapy techniques persists as conventional medications present deleterious side effects. This study showed that *L. plantarum* L15 prevented DSS-induced colitis by reducing pro-inflammatory cytokines (TNF-α, IL-1β, IL-6) and elevating anti-inflammatory cytokine(IL-10)level, up-regulating the mRNA expression level of tight junction protein (ZO-1, Occludin, Claudin-1) and mucin (MUC2), and decreasing D-lactic acid and LPS. Its supplementation can also modulate the gut microbial structure and increase SCFA production (acetate and butyrate), further inhibiting the NF-κB signaling pathway. This study suggested that *L. plantarum* L15 can be used as probiotics for preventing UC.

## Data availability statement

The data presented in the study are deposited in the NCBI repository (https://www.ncbi.nlm.nih.gov/sra/), accession number PRJNA861930.

## Ethics statement

The animal study was reviewed and approved by the Northeast Agricultural University Animal Care approved all animal experiments and protocols (Authorization No: NEAUEC2001121).

## Author contributions

BL and ZG designed the study. ML and YG performed the experiments. ZW, QC, and LY wrote the manuscript. HT, KZ, and CL analyzed the data. All authors contributed to the article and approved the submitted version.

## Funding

This research was funded by the Present research work was financially supported by the National Natural Science Foundation of China (32101919), Young Elite Scientist Sponsorship Program by CAST (YESS20200271), the Natural Science Foundation of Heilongjiang Province (YQ2020C013), Heilongjiang Postdoctoral Science Foundation (LBH-TZ2103 and LBH-Z20006), Heilongjiang Province Hundred Million Engineering Science and Technology Major Project (2021ZX12B02), Key R&D projects in Heilongjiang Province (GY2021ZB0204).

## Conflict of interest

The authors declare that the research was conducted in the absence of any commercial or financial relationships that could be construed as a potential conflict of interest.

## Publisher’s note

All claims expressed in this article are solely those of the authors and do not necessarily represent those of their affiliated organizations, or those of the publisher, the editors and the reviewers. Any product that may be evaluated in this article, or claim that may be made by its manufacturer, is not guaranteed or endorsed by the publisher.
