# Supplementary material for: In vivo evidence of the prevents DSS-induced colitis of Lactiplantibacillus plantarum L15
Source: Front Microbiol. 2022 Oct 6;13:1028919. doi: 10.3389/fmicb.2022.1028919 (PMC9583153; doi:10.3389/fmicb.2022.1028919)
Supplement: Supplementary file 1 [file Data_Sheet_1.ZIP › Raw Data8.25/Table.docx]

**Table**

**Table 1** Primer sequences for qPT- PCR.

| **Genes** | **Forward（5’-3’)** | | **Reverse（5’-3’)** |
| --- | --- | --- | --- |
| Claudin1 | | GCTGGGTTTCATCCTGGCTTCTC | CCTGAGCGGTCACGATGTTGTC |
| Occludin | | TTGGCTACGGAGGTGGCTATGG | TTACTAAGGAAGCGATGAAGCAGAAGG |
| ZO-1 | | CATAAGGAGGTAGAACGAGGCATCATC | CGATCACCACCCGCTGTCTTTG |
| β-actin | | GGTTGTCTCCTGCGACTTCA | TGGTCCAGGGTTTCTTACTCC |
